# The Genetic Basis for Salivary Gland Barriers to Arboviral Transmission

**DOI:** 10.3390/insects12010073

**Published:** 2021-01-15

**Authors:** Irma Sanchez-Vargas, Ken E. Olson, William C. Black

**Affiliations:** Center for Vector-borne Infectious Diseases, Department of Microbiology, Immunology, and Pathology, Colorado State University, Ft. Collins, CO 80523, USA; irma.sanchez-vargas@colostate.edu (I.S.-V.); william.black@colostate.edu (W.C.B.IV)

**Keywords:** *Aedes aegypti*, arbovirus, salivary gland infection barriers, salivary gland escape barriers, quantitative genetics

## Abstract

**Simple Summary:**

Mosquito salivary glands are infected with an arbovirus prior to entering saliva. The virus in the mosquito’s saliva can then infect a vertebrate host when the vector acquires its next bloodmeal. Salivary gland infection and escape barriers (SGIB and SGEB, respectively) have been identified that modulate arbovirus transmission. SGIB are manifest as the absence of virus in the salivary glands of mosquitoes having a disseminated infection, while SGEB are evident as an absence of virus in the saliva of mosquitoes even though salivary glands are infected. The interaction between *Aedes aegypti* and viruses is dynamic and related to the genetic background of each vector population and virus variant. There is little understanding of the genetic basis for SGIB and SGEB. Here, nested, half-sib families of *Aedes aegypti* were used to estimate genetic and environmental variances, in which daughters from an individual dam differ in their phenotype due to the sire. We found that SGIB has a strong genetic basis with dengue virus infections but not with Zika or chikungunya virus infections, in which all salivary glands became infected. SGEB has a moderate genetic basis for Zika or chikungunya infections but not with dengue virus infections.

**Abstract:**

Arthropod-borne viruses (arboviruses) infect mosquito salivary glands and then escape to saliva prior to virus transmission. Arbovirus transmission from mosquitoes can be modulated by salivary gland infection barriers (SGIBs) and salivary gland escape barriers (SGEBs). We determined the influence of SGIBs and SGEBs by estimating the quantitative genetic contributions of *Aedes aegypti* half-sib families (Mapastepec, Mexico) infected with three dengue 2 (DENV2), two chikungunya (CHIKV), and two Zika (ZIKV) genotypes. We determined virus titer per salivary gland and saliva at seven days post-infection and virus prevalence in the half-sib population. CHIKV or ZIKV genotypes did not present SGIB, whereas DENV2 genotypes showed low rates of SGIB. However, virus titer and prevalence due to additive genetic factors in the half-sib family displayed a significant narrow-sense heritability (h^2^) for SGIB in two of the three DENV2 genotypes and one CHIKV and one ZIKV genotype. SGEBs were detected in all seven virus strains: 60–88% of DENV2 and 48–62% of CHIKV or ZIKV genotype infections. SGEB h^2^ was significant for all CHIKV or ZIKV genotypes but not for any of the DENV2 genotypes. SGIBs and SGEBs exhibited classical gene-by-gene interaction dynamics and are influenced by genetic factors in the mosquito and the virus.

## 1. Introduction

**Figure 1:**
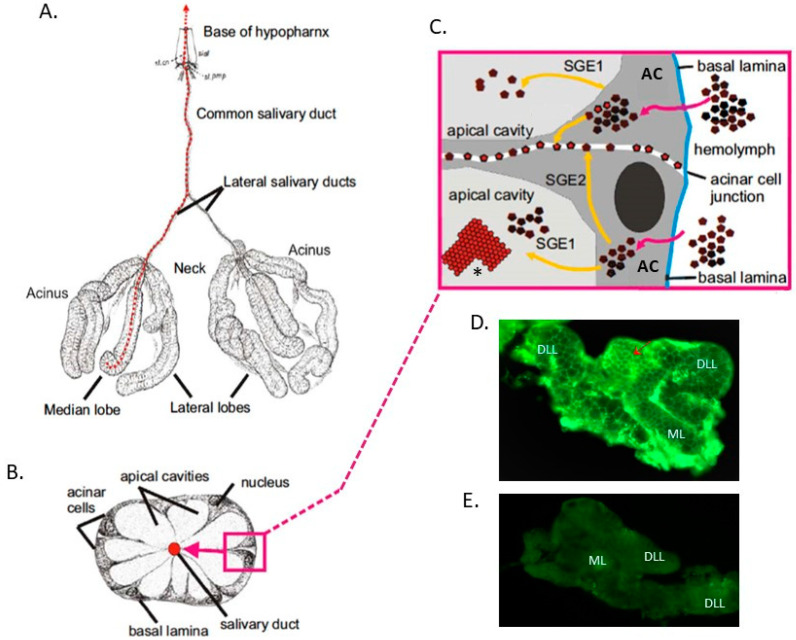
Structure of *Aedes* spp. salivary glands [3]. (**A**) Schematic representation of salivary glands of a mosquito at 2 days post-emergence. (**B**) Transverse section through a median lobe of the salivary gland showing the acinar cells and their relationship to the salivary duct. (**C**) An enlargement of B showing a cartoon of a transverse-section of salivary gland indicating mature virus penetrating the basal lamina to establish an infection in the acinar cells (**A**,**C**) and then penetrating the plasma membrane to fill the apical cavity and/or accumulate in the acinar cell junctions. Pink arrows trace arbovirus entry and movement in individual acinar cells leading to the virus’ eventual release through the apical cavity (yellow lines). Salivary gland escape (SGE)1 and SGE2 refer to potential barriers (entry into the apical cavity and acinar cell junctions, respectively) for virus escape from salivary glands (**D**) Immunofluorescence of salivary gland lobe infected with DENV-2 Merida (Cosmopolitan) at 7 dpi shows that mature viral particles clearly accumulated in acinar cell junctions (red arrow). (**E**) Uninfected salivary glands showed no immunofluorescence signal. N: nuclei. SD: salivary duct. AC: apical cavity for saliva storage. BL: basal lamina. Cyt: Cytoplasm. CM: Cell membrane. DLL: distal lateral lobe, ML: medial lobe. * Represents a virus cluster (1C).

Vector competence (VC) for an arbovirus is affected by intrinsic (genetic) factors and mechanisms that control the ability of a vector mosquito to acquire, maintain, and transmit an arbovirus. There are several tissue barriers in the mosquito that the virus must overcome in order to establish a productive infection: the midgut infection barrier (MIB), midgut escape barrier (MEB), salivary gland infection barrier (SGIB), and salivary gland escape barrier (SGEB). In mosquito vectors of disease, the salivary gland is the organ leading to transmission of an arbovirus from an infected mosquito [1]. In all mosquitoes, the two salivary glands are located in the anterior portion of the thoracic hemocele. In females, each gland consists of two lateral and one medial cylindrical lobe (Figure 1A). The distal end of each lobe is called the acinus, while the proximal end is called the neck (Figure 1A). Each lobe consists of a basal lamina bounding a single layer of acinar cells (Figure 1B). These cells are distributed around a central salivary duct containing an apical cavity for saliva storage. Apical plasma membranes have microvillar projections that are highly convoluted to increase the surface area for discharge of saliva into the apical cavity (diverticulum) (Figure 1B). Following successful dissemination from the midgut, arboviruses typically infect hemocytes, fat body, neural tissue, and occasionally muscle tissue before infecting the salivary glands. Virus amplification in fat body and specifically in hemocytes is an important prerequisite before the virus is ready to infect the salivary glands [2]. Virus in the hemolymph penetrates the basal lamina of the salivary glands surrounding acinar cells to generate a salivary gland infection (Figure 1C) [3]. Factors that prevent virus penetration of the salivary gland basal lamina constitute SGIB. Several studies suggest that tracheoles or nerve tissues may allow virus entry into acinar cells [4,5], where viral replication occurs. Barriers that prevent infection and virus replication in the salivary glands also constitute SGIB (SGI, Figure 1C). Viruses are eventually released in the apical cavities where saliva is stored prior to its release into the hypopharynx and deposition of virus into a vertebrate during probing and feeding. Barriers that block release of mature virus in the apical cavities are referred to as SGE1 and SGE2 (Figure 1C). Virus replication in the salivary glands has been reported for DENV [6], CHIKV [3], and ZIKV [7].

The existence of SGEB has been definitively demonstrated for Japanese encephalitis virus (JEV) in *Culex tritaeniorhynchus* [8], Snowshoe hare virus in *Aedes triseriatus* [9], La Crosse virus (LACV) in *Aedes hendersoni* [10], and Sindbis virus (SINV) in *Culex theileri* [11]. An exceptionally strong SGEB was detected in *Ae. hendersoni* for LACV, wherein 65% of females had salivary gland infections by plaque assay, but only 5% actually transmitted infectious virus [12]. These results were confirmed by immunofluorescence assays (IFA), which showed high concentrations of LACV antigens in the salivary glands. More recently, SGEB have been reported for Rift Valley fever virus (RVFV) in *Culex nigripalpus, Anopheles crucians,* and *Aedes infirmatus* [13,14]. A recent transmission electron microscopy study [3] nicely illustrated CHIKV replication and storage in *Ae. albopictus* salivary glands and showed that CHIKV nucleocapsids bud from two different sites in acinar cells to become mature particles. The first site is at vesicular membranes located in the apical cavity of acinar cells (SGE1, Figure 1C), while the second occurs at cellular plasma membranes (SGE2, Figure 1C). Mature viral particles clearly accumulated in acinar cell junctions (Figure 1D) [3].

Furthermore, it has been shown that different viruses preferentially replicate in different lobes of the salivary glands [15]. Arbovirus infection of salivary glands typically begins in the distal lateral lobes [15,16,17,18,19]. Arboviruses such as DENV2 and CHIKV infect the proximal lateral and medial [20] lobes of *Ae. aegypti*, whereas SINV does not seem to infect the median lobes of *Aedes albopictus* or *Ae. aegypti* [15,16,20,21]. Electron micrographic studies confirm that clumps of crystalline flavi- and alphaviruses [3,22] occur in the salivary glands and saliva. It has been reported that the lateral and medial salivary gland lobes interact with monoclonal antibodies differently, suggesting there is a biochemical difference between these two lobe surfaces [23]. Others have observed that specific proteins are differentially expressed by different salivary gland lobes [24], which could account for the observed differences between lobes or between mosquitoes in susceptibility to virus infection [2,25].

To reach the salivary glands, arboviruses must overcome natural barriers, infect various tissues, exploit physical conduits, and counteract innate and cellular defenses [15,26,27,28]. Meanwhile, vectorial capacity involves extrinsic factors, including temperature, availability of vertebrate hosts, feeding behavior, population density, longevity, and predation [29]. The interaction between *Ae. aegypti* and a virus is dynamic and is related to the genetic background of each particular vector population and arbovirus variant [30,31,32,33]. It is possible that the existence of mosquito genetic variants is related to these functions, which could affect the degree of susceptibility in mosquitoes [34]. There is little understanding of whether there is a genetic basis for SGIB and SGEB or whether they are random and subject to a few or many of the “environmental” factors listed in the previous paragraph. One of the principal applications of quantitative genetics is to determine the relative contributions of genetic and environmental factors to variation and covariation in one or more quantitative phenotypes [35]. Basically, the phenotypic variance (Vp) of a population is viewed as the sum of separate genetic (Vg) and environmental (Ve) variances. The genetic variance is in turn made up of additive (Va) and non-additive (dominance/epistatic) components. Va and the associated narrow-sense heritability (h^2^ = Va/Vp) can vary from 0 to 1, and indicate the proportion of phenotypic variance explained by additive genetic effects [35].

Herein, we assume that if the variances in SGIB and SGEB among siblings or between half-sib families are due to one or all of the random causes listed above, including variation in the amount of saliva produced by a female, then Ve will be large, Va will be small, and h^2^ will approach zero. Alternatively, if the amount of virus collected in saliva is due primarily to genetic, rather than environmental, factors, then the variation in SGIB and SGEB will have a large h^2^, approaching one. Herein, we perform a half-sib analysis to estimate the quantitative genetic and environmental basis of SGIB and SGEB for three DENV2 genotypes (American, Cosmopolitan, and Asian genotype), two CHIKV genotypes (Asian and ECSA-IOL genotype) and two ZIKV genotypes (Asian and African genotype). Basically, we are testing if, given all the environmental effects listed above, a significant genetic effect can be detected.

## 2. Materials and Methods

### 2.1. Cells, Viruses, and Mosquitoes

C6/36 cells (ATCC CRL-1660), Vero (ATCC CCL-81), and LLC-MK2 cells (ATCC number CCL-7.1) were maintained in minimum essential medium (MEM) supplemented with 7% heat-inactivated (56 °C, 30 min) fetal bovine serum (FBS, from Atlas Biologicals, cat# F-0500-A, Fort Collins, CO, USA), 1% L-glutamine solution (Glutamine from Mediatech, Corning catalog# 25-005-Cl, Tewksbury, MA, USA), 1% non-essential amino acids solution (Nonessential amino acids from Mediatech 25-025-Cl), and 1% penicillin/streptomycin (Pen-Strep from Mediatech, catalog# 30-001-Cl). The cells were incubated at 37 °C, 5% CO_2_. Virus isolates used in this study include DENV2 Quintana Roo 94 (Qro94, American genotype; Accession# DQ341196), DENV2 Merida BC17 (Cosmopolitan genotype; Accession# AY449677), DENV2 Guerrero C932 (Asian II genotype; Accession# AY449678), CHIKV R99659 (Asian genotype; Accession# KJ451624.1), CHIKV LR2006 OPY-1 (La Réunion, ECSA-IOL genotype; Accession # DQ443544.2), ZIKV PRVABC59 (Asian genotype; Accession# KU501215), and ZIKV Dakar 41525 (African genotype; Accession# KU955591). Hereafter, viruses are designated in the text as DENV2 American, DENV2 Cosmopolitan, DENV2 Asian, CHIKV Asian, CHIKV ECSA-IOL, ZIKV Asian, and ZIKV African, respectively. DENV2 isolates were endemic strains from Mexico. The virus strains used in this study have different levels of vector competence: low for DENV2 American, moderate for DENV2 Asian, CHIKV Asian, and ZIKV Asian, and high for DENV2 Cosmopolitan and ZIKV African. The American, Cosmopolitan, and Asian DENV2 isolates used here were available in our lab and afforded us the ability to determine heritability in the context of three DENV2 genotypes from Mexico. DENV2 were propagated in C6/36 cells at a multiplicity of infection (MOI) of 0.01. ZIKV and CHIKV were propagated similarly in Vero cells. Supernatant was harvested (3–5 days post-infection (dpi) for CHIKV and ZIKV and 10–12 dpi for DENV), clarified by centrifugation at 4 °C, and aliquoted into single-use vials before freezing at −80 °C. *Aedes aegypti* strain Mapastepec (15°26′44.05′′ N, 92°54′9.74′′ W), a domestic mosquito from an urban region in from the state of Chiapas, Mexico, was originally collected in 2016. Adult mosquitoes were fed on raisins and water, and the females were allowed to feed on uninfected blood meals to stimulate oogenesis. Mosquitoes were maintained at 28 °C, 80% relative humidity with 16–8 h light-dark periods. Half-sib families for this study were created using generations F4, F5, and F6.

### 2.2. Intrathoracic Inoculation

Intrathoracic injections of DENV, ZIKV, and CHIKV were conducted to assess virus replication in salivary glands and other mosquito tissues outside the midgut. Intrathoracic inoculation of mosquitoes with arboviruses usually leads to more rapid, and higher proportions of, disseminated infections, compared to oral challenge, and mosquitoes become infected with a constant dose of virus [14,15,36,37]. We acknowledge that intrathoracic injections bypass the midgut, arguably the most important barrier to mosquito infection. However, past experience [15] has shown that only a few orally-infected mosquitoes in a family actually go on to develop infected salivary glands. These small numbers preclude an accurate quantitative genetic analysis of SGIB and SGEB. Notably, the peak concentration of virus detected in the salivary glands is often similar, regardless of which method is initially used [15]. The method for intrathoracic inoculation of mosquitoes is described in [38] with some modifications. Adult females (7 days post-eclosion) were infected by injecting 500 plaque forming units (PFU) in a volume of 69 nL of the virus stock using a Nanojet II (Drummond Scientific Company, Broomall, PA, USA). All injections were performed under a dissecting microscope using glass needles that were prepared using a vertical pipette puller (P-30, Sutter Instrument Co., Novato, CA, USA). Mosquitoes were left for 7 days at 28 °C and 80% relative humidity before samples were collected.

### 2.3. Saliva Collection and Salivary Gland Dissections

**Figure 2:**
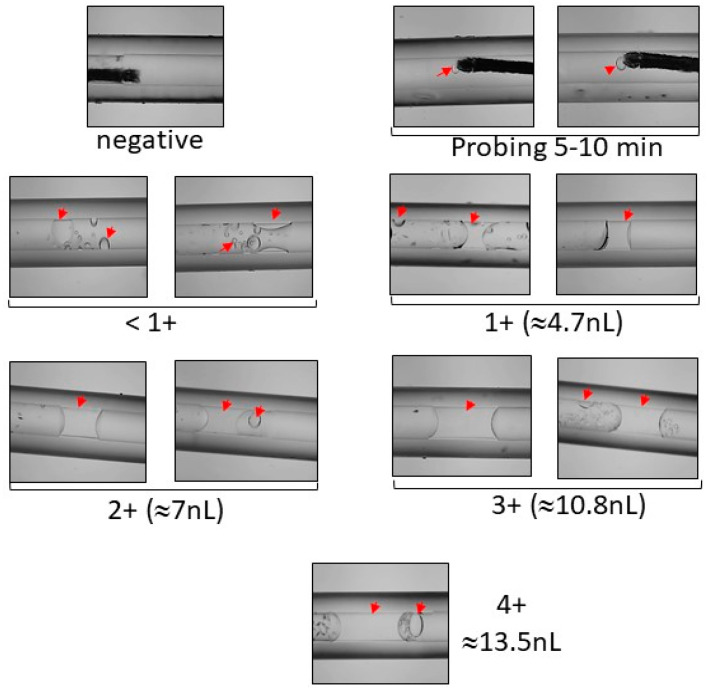
Individual capillaries observed under the microscope for the presence of saliva and assigned saliva categories based on total volume. The volume of saliva was calculated using the cylinder volume formula (V = π (r^2^ × h) = 3.1416 × 0.01 × h = mm^3^ or µL) and measuring the height of the saliva with a digital fractional caliper (precision: ±0.02 mm), as described previously.

At 7 dpi, saliva from each of 10–15 daughters/dam were collected [39]. Briefly, females were chilled, and their wings and legs removed. Their proboscises were inserted into a 1 µL micropipette (microcaps^®^, Drummond Scientific Company, PA, USA) filled with immersion oil type B, and the mosquitoes were allowed to salivate into the oil at room temperature. After 5 min (probing) and 45–60 min, each individual capillary was observed under the stereoscope Olympus SZH10 at 2X magnification for the presence of saliva. Mosquitoes that did not visibly salivate were eliminated. To determine the relationship between saliva volume and saliva titer, categories were assigned 1 (≈4.7 nL), 2 (≈7 nL), 3 (≈10.8 nL), or 4 (≈13.5 nL), based on the total observed volume compared with a reference capillary (Figure 2) [40].

The proboscis was removed from the capillary, and oil containing the saliva was expelled under pressure into 1.5 mL tubes containing 300 μL Dulbecco’s Modified Eagle Medium (DMEM supplemented with 20% of heat inactivated FBS, 1% penicillin/streptomycin, 1% glutamine, 1% non-essential amino acids) and frozen immediately on dry ice. After collecting saliva, salivary glands from mosquitoes were dissected and collected in 500 μL DMEM medium (7% heat inactivated FBS, 1% penicillin/streptomycin, 1% glutamine, 1% non-essential amino acids) and frozen immediately on dry ice. Virus in saliva and salivary glands were detected and quantified by plaque assay.

### 2.4. Infectious Virus Titration by Plaque Assay

We used LLC-MK2 cells for DENV2 plaque assays and Vero cells for ZIKV and CHIKV plaque assays. Plaque assays were performed on confluent monolayer cells in 24-well plates, infected with 10-fold serial dilutions of saliva samples or salivary gland homogenate that had been sterile-filtered with Acrodisc syringe filters with Supor membrane 0.2 μm (Pall Life Sciences, East Hills, NY, USA). After 1 h, infected cells were overlaid with a 1% agar–nutrient mixture [agar solution (1 g/79 mL ddH2O): nutrient solution (10 mL of 10×/100 mL Media 199, 5% of heat inactivated FBS, 7.5% sodium bicarbonate 4 mL/100 mL), 2% DEAE-dextrose in Hanks balanced solution (1 mL/100 mL), 0.5 mL/100 mL MEM essential amino acids (15× solution), and 0.5 mL/100 mL MEM Vitamins (100× solution)]. After 4, 6, or 10 days incubation at 37 °C (CHIKV, ZIKV, and DENV2 respectively), cells were stained with 150 µL/well of 3 mg/mL MTT (3-[4,5-dimethylthiazol-2-yl]-2,5-diphenyltetrazolium bromide) solution and incubated for at least 4 h [41,42]. Viral titers were determined by counting plaques. Individual saliva or salivary gland titers are reported as plaque forming units per milliliter (PFU/mL). All virus negative (saliva or salivary gland) samples were tested twice to be certain they were really negative. The limit of detection for the plaque assays was 1 PFU/mL for CHIKV and ZIKV virus and 1–10 PFU/mL for DENV.

### 2.5. Quantitative Genetic Analyses

**Figure 3:**
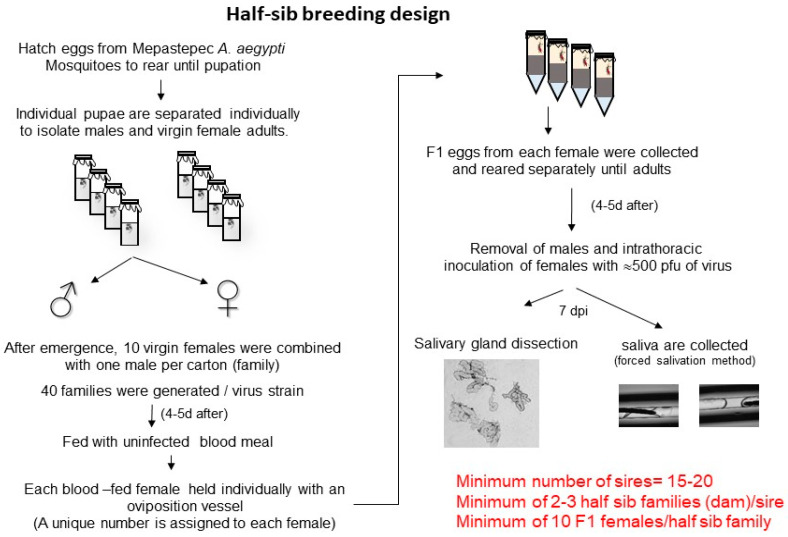
Method for generating nested half-sib design used for calculating estimates of genetic and environmental variances and covariances.

A nested half-sib design was used to provide estimates of genetic and environmental variances and covariances [35,43,44]. A single male was mated to several (usually 3–5) females. The females were allowed to bloodfeed and lay eggs. Eggs from each female were collected, and 5 days later, were reared separately, awaiting adult eclosion (Figure 3). All 7-day-old daughters from an individual dam were intrathoracically inoculated with 500 PFU of virus, and after 7 days, were tested for the presence or absence of virus and the amount of virus in the salivary glands and saliva (Figure 3). Half-sib families differ in their phenotype due to the sire. If the SGIB or SGEB have a genetic basis, then sharing the same sire is why half-sibs are similar for the SGIB or SGEB phenotype. Variance in phenotype between half-sib families is equal to the covariance in phenotype among half-sibs. A high variance between families should cause high covariance within families, because members of a family are alike and the heritability approaches one. Conversely, a low variance in phenotype between families will lead to low covariance within families, and the heritability approaches zero.

Restricted maximum likelihood (REML) was used to estimate quantitative genetic parameters [44,45]. REML does not require balanced designs, and missing cells have a minimal effect. Furthermore, REML allows a diversity of hypotheses to be tested. The computational limits that formerly made REML impractical for large datasets have mostly been overcome. For these reasons, REML is now the method of choice for estimating quantitative genetic parameters from most designs. We used PROC MIXED in SAS (SAS User’s Guide, Cary, NC: Statistical Analysis System Institute, Inc., 1987) to obtain REML estimates and hypothesis tests for half-sib designs [44,45]. The SAS code for performing REML analyses appears in Figure S1. The total phenotypic variance (σ^2^_P_) is a function of three observed sources of variance: the average variance among offspring of a single sire is σ^2^_S_, the average variance among full-sib families from dams mated to a common sire is σ^2^_d_, and the average variance within members of a full-sib family is σ^2^_w_. Additive genetic variance (Va) is calculated as:

4 σ^2^_S_ (1)

The variance among dams (Vd) bred to a common sire is calculated as:

Vd = σ^2^_d_ − σ^2^_S_ (2)

The variance among full-sibs is (Vw):

Vw = σ^2^_w_ − 2 σ^2^_S_ (3)

Summing Equations (1)–(3),

Vp = Va + Vd + Ve = 4 σ^2^_S_ + (σ^2^_d_ − σ^2^_S_) + (σ^2^_w_ − 2 σ^2^_S_) = σ^2^_S_ + σ^2^_d_ + σ^2^_w_ (4)

Narrow-sense heritability (h^2^) is:

h^2^ = Va/Vp (5)

See SAS lines 5–10 for SGIB, and lines 24–30 for SGEB in Figure S1. The null hypothesis that σ^2^_S_ = 0 was analyzed with a likelihood ratio test. The 2 restricted log likelihood for the full model (Equation (4)) was subtracted from 2 restricted log likelihood for the Va = 0 mode

Vp = Vd + Ve(6)

See SAS lines 12–23 for SGIB and lines 31–42 for SGEB in Supplement 1. This is a likelihood ratio test because the difference in log likelihoods equals the log of the ratio of the likelihoods. There is one degree of freedom (d. f.) because Equations (4) and (6) differ by one parameter.

### 2.6. Statistical Analyses

All statistical analyses were performed with GraphPad Prism (version 5.0, La Jolla, CA, USA). A two-tailed Fisher’s exact test was used to compare infection rates for SGIB and SGEB and determine any correlation among virus titers in saliva and salivary glands (Pearson correlation). Analysis of variance (one-way and two-way ANOVA) was used to determine the statistical significance, and CORREL function (Excel 2016, Microsoft, Redmond, DC, USA) was used for determining the correlation coefficient. Significance was defined as *p* < 0.05.

## 3. Results

### 3.1. Infection Rates

Our breeding design was established with a minimum number of sires (15–20), a minimum of 2–3 half-sib families (dam/sire) and a minimum of ten F1 females/half-sib families. Under this criterion, we obtained 24 half-sib families for DENV2 American genotype. We also established 21 half-sib families for DENV2 Asian and Cosmopolitan genotypes, ZIKV Asian and African genotypes, and 22 half-sib families for CHIKV genotypes. Table 1 lists the number of paired samples tested for each of the seven viral strains. The number of salivary glands that were not infected divided by the total number of salivary glands tested is the SGIB%. The number of infected salivary glands that failed to produce any virus in the saliva are also listed. This number divided by the number of infected salivary glands is the SGEB%. The percentage transmission is 100 − SGEB% (Table 1).

**Figure 4:**
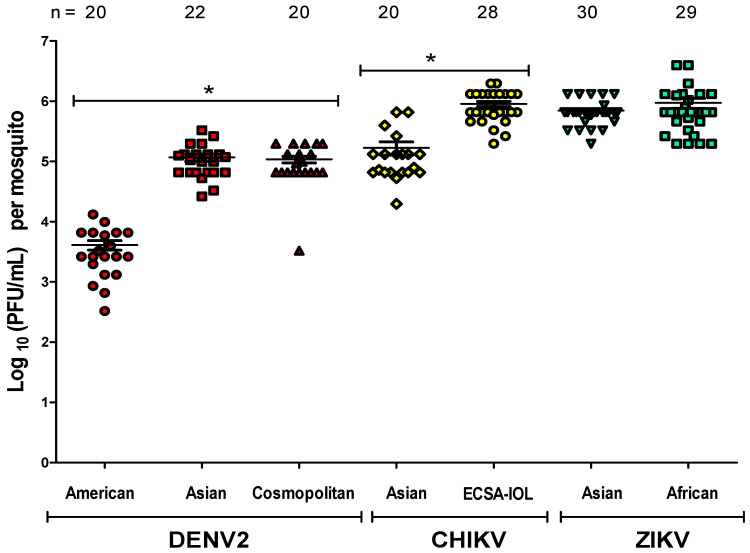
Titer of Mapastepec *Aedes aegypti* mosquitoes 7 days after intrathoracically injecting with 500 plaque forming units (PFU) of virus. Titers were determined by plaque assay in the whole mosquito for each viral strain. Means and 95% confidence intervals are shown. * = *p* < 0.0001.

These calculations assume that viral dissemination occurred in each intrathoracically-inoculated mosquito based on data that shows 100% infection of whole mosquitoes (*n* = 20–30) by plaque assay for each viral strain at 7 dpi (Figure 4).

Dissemination rates after intrathoracic inoculation were typically between 95–100% [46]. DENV2 American virus titer was significantly lower than other DENV genotypes as well as CHIKV and ZIKV titers (*p* < 0.0001). Significant differences were also detected in virus titer between CHIKV genotypes (*p* < 0.0001). CHIKV ECSA-IOL virus titers were higher (9 × 10^5^ ± 9 × 10^4^ PFU/mL) than CHIKV Asian virus titers (1.7 × 10^5^ ± 1.9 × 10^5^ PFU/mL). We observed no significant differences in virus titer between the two ZIKV genotypes (*p* = 0.8931) or between DENV2 Asian and Cosmopolitan genotypes (*p* = 0.6971) (Figure 4).

### 3.2. Salivary Gland Infection Barrier (SGIB)

**Figure 5:**
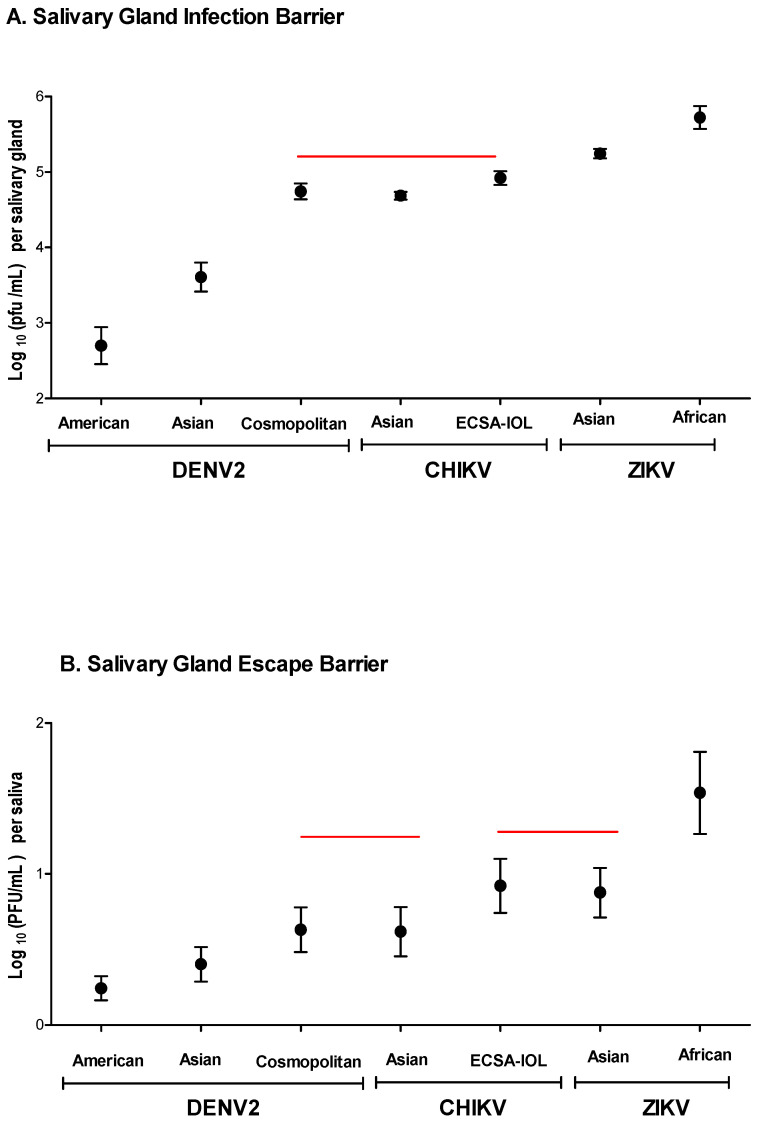
(**A**) Average numbers of log_10_ (PFU/mL) per salivary gland for all seven viral genotypes. (**B**) Average numbers of log_10_ (PFU/mL) per saliva were calculated for all seven viral genotypes. Horizontal lines over pairs of genotypes in either graph indicate that average log_10_ (PFU/mL) per salivary gland did not differ significantly.

CHIKV or ZIKV genotypes did not present SGIB, whereas DENV2 genotypes showed low rates of SGIB (Table 1). Specifically, after 7 dpi, 5.5% (38/691), 0.75% (5/667), and 0.59% (4/681) of *Ae. aegypti* salivary glands were uninfected with the DENV2 American, Asian, and Cosmopolitan genotypes, respectively. All salivary glands, on the other hand, were infected with CHIKV or ZIKV. Average viral titer (log_10_ (PFU/mL)) per salivary gland was calculated for all seven virus genotypes. Means and 95% confidence intervals are shown in Figure 5A.

**Figure 6:**
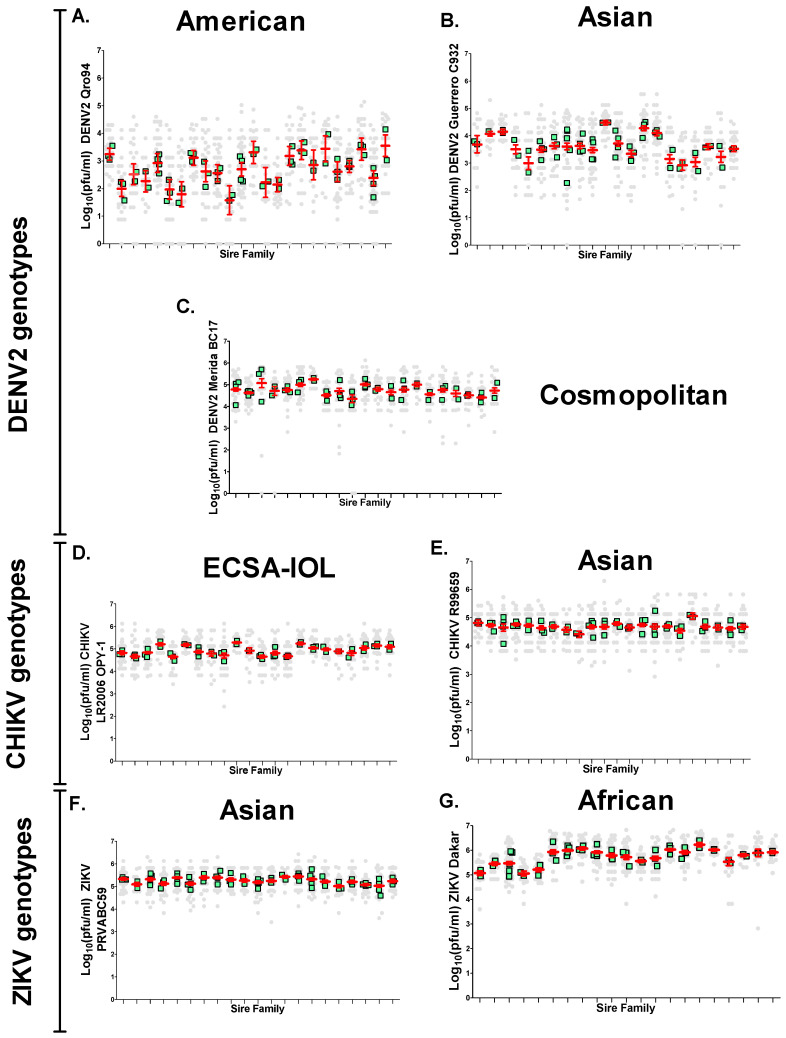
Distribution of phenotypes log_10_ (PFU/mL) per salivary gland in half-sib families for the seven virus strains. Each column of points arising from the *x*-axis represents a sire family. In each column, the distribution of individual salivary glands titers arising from that sire appear as gray circles. Mean sire values and their 95% confidence intervals are displayed in red. The mean of dam families in each sire appear in green. (**A**) DENV2 American genotype, (**B**) DENV2 Asian genotype, (**C**) DENV2 Cosmopolitan genotype, (**D**) CHIKV ESCA-IOL genotype, (**E**) CHIKV Asian genotype, (**F**) ZIKV Asian genotype, and (**G**) ZIKV African genotype.

When comparing genotypes across the same virus family (DENV2, CHIKV, and ZIKV), genotype titer means for DENV2 and ZIKV were significantly different (*p* < 0.0001). Of all viruses tested, DENV2 American had the lowest virus titer (2.699 ± 0.58 log_10_ (PFU/mL)), while ZIKV African had the highest titer (5.723 ± 0.331 log_10_ (PFU/mL)). DENV2 Cosmopolitan had the highest virus titer (4.742 ± 0.23 log_10_ (PFU/mL)) between DENV2 genotypes, while DENV2 Asian (3.607 ± 0.0421 log_10_ (PFU/mL)) was the second lowest of all viruses tested. CHIKV ECSA-IOL had slightly higher titers (4.921 ± 0.206 log_10_ (PFU/mL)) as compared to CHIKV Asian (4.687 ± 0.711 log_10_ (PFU/mL)). CHIKV genotypes had the same titers as DENV2 Cosmopolitan (*p* = 0.3294). Both ZIKV genotypes (Asian, 5.244 ± 0.136 log_10_ (PFU/mL); African, 5.723 ± 0.331 log_10_ (PFU/mL)) exhibited the highest titers. SGIB did affect the quantity of virus present in the salivary gland. It is very clear that different half-sib families can, and do, vary greatly in the amount of virus in salivary glands (Figure 6).

When comparing the spread of sire means between DENV2 genotypes, the greatest variation in log_10_ (PFU/mL) means/sire occurred with DENV2 American (Figure 6A) infection, and the smallest spread of sire means occurred with DENV2 Cosmopolitan infection (Figure 6C). Variation among sire families with CHIKV ECSA-IOL (Figure 6D) was slightly greater than that among CHIKV Asian families (Figure 6E). Variation among sire families with ZIKV Asian and ZIKV African were also small (Figure 6F,G). Narrow-sense heritabilities (h^2^) for SGIB were calculated to characterize each viral infection (Table 2) once the observational components σ^2^_S_, σ^2^_d_, and σ^2^_w_ and the causal components of variance (Vp, Vsire, Vdam, Ve) for log_10_ (PFU/mL)/salivary gland were obtained (Table S1).

SGIB exhibited strong genetic bases with DENV American and Asian genotypes, but not with DENV2 Cosmopolitan genotype. For DENV2 American genotype and DENV2 Asian genotype, both the estimate of the variance among sire means and the h^2^ were significantly different (for American: σ^2^_S_ = 0.2700, *p* = 0.0028; h^2^ = 0.8103, *p* = 5.34 × 10^−7^; for Asian: σ^2^_S_ = 0.1029, *p* = 0.0348; h^2^ = 0.5799, *p* = 0.0072). However, for DENV2 Cosmopolitan genotype, the variance among sire means were small (0.0082) and non-significant (*p* = 0.3238). The narrow-sense heritability h^2^ was 0.0685 (*p* = 0.3274).

There were large differences between the CHIKV and ZIKV genotypes. SGIB exhibited strong genetic bases with CHIKV ECSA-IOL and ZIKV African, but not CHIKV or ZIKV Asian genotypes. For CHIKV ECSA-IOL and ZIKV African, the variance among sire means and the h^2^ were significantly different (for CHIKV ECSA-IOL: σ^2^_S_ = 0.0337, *p* = 0.005; h^2^ = 0.5174, *p* = 6.2 × 10^−6^; for ZIKV African: σ^2^_S_ = 0.0762, *p* = 0.0133; h^2^ = 0.3106, *p* = 0.0009). For CHIKV Asian genotype, the variance among sire means and the h^2^ were not significantly different (σ^2^_S_ = 0.0012, *p* = 0.4537; h^2^ = 0.0385, *p* = 0.5). For ZIKV Asian genotype, the variance among sire means was 0.008 and not significant (*p* = 0.0918), and h^2^ was 0.1526 and marginally significant (*p* = 0.0416). There was a positive correlation between the variance among sire means and the narrow-sense heritability (h^2^). As predicted by Equation (5) (h^2^ = Va/Vp), the greater the variance in sire means, the larger the heritability. In other words, when the amount of virus was low (e.g., Figure 6A,B, DENV2 American and Asian), estimates of heritability and variance among sire means tended to be large and significant. However, once viral titers reach a high enough level in the salivary gland, (DENV2 Cosmopolitan, CHIKV, and ZIKV genotypes in Figure 6C–G), genes in the mosquito may no longer have an effect, and heritability estimates were small.

**Figure 7:**
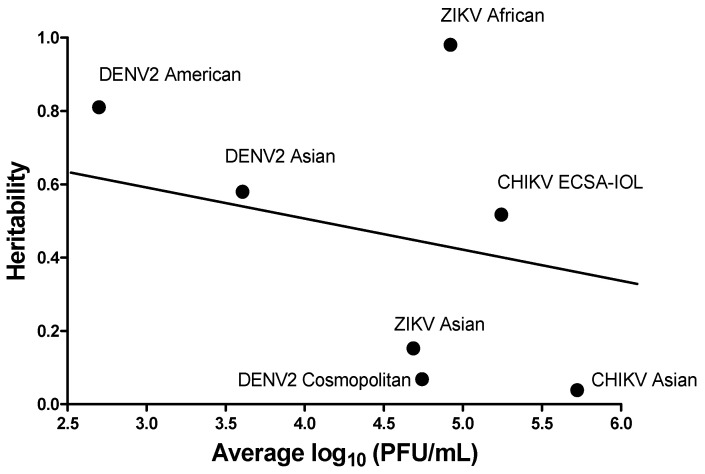
Correlation analysis to test whether the average log_10_ (PFU/mL) virus in the salivary glands are negatively correlated with the magnitude of heritability.

Variation among sire families (Figure 6) suggested that viral titer in the salivary gland is negatively correlated with the magnitude of heritability. We performed a correlation analysis between the average log_10_ PFU/mL virus in the salivary glands and the heritability to evaluate this observation (Figure 7). Pearson’s correlation coefficient was large and negative, as expected, when ZIKV African was excluded (R = −0.819, *p* = 0.0461). If ZIKV African was included, Pearson correlation coefficient was −0.24150 (*p* = 0.6019).

### 3.3. Salivary Gland Escape Barrier

In contrast to SGIB, all seven virus strains showed SGEB (Table 1). SGEB% (uninfected saliva/infected salivary glands) rates for DENV2 genotypes ranged, from 88.5% (578/653), 72.5% (480/662), 59.7% (404/677) for American, Asian, and Cosmopolitan, respectively. CHIKV genotypes showed 47.7% (327/686) and 61.5% (452/735) for ECSA-IOL and Asian, respectively. Finally, we obtained 52.1% (408/783) for ZIKV Asian and 32.1% (200/623) for ZIKV African. All three DENV2 genotypes varied significantly, as did the two CHIKV genotypes (*p* < 0.0001). DENV2 Cosmopolitan and CHIKV Asian did not significantly differ in SGEB% (*p* = 0.9447), nor did CHIKV ECSA-IOL or ZIKV Asian (*p* = 0.7332).

**Figure 8:**
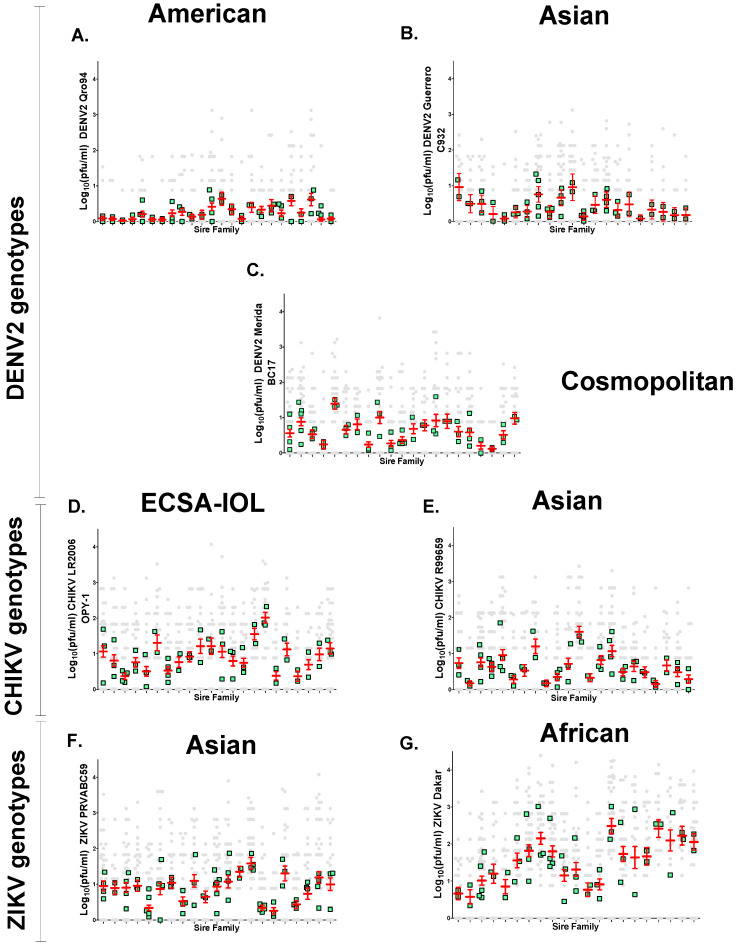
Distribution of phenotypes log_10_ (PFU/mL) per saliva in half-sib families for the seven virus strains. Each column of points arising from the X-axis represents a sire family. In each column, the distribution of individual saliva titer arising from that sire appear as gray circles. Mean sire values and their 95% confidence intervals are displayed in red. The mean of dam families in each sire appear in green. (**A**) DENV2 American genotype, (**B**) DENV2 Asian genotype, (**C**) DENV2 Cosmopolitan genotype, (**D**) CHIKV ESCA-IOL genotype, (**E**) CHIKV Asian genotype, (**F**) ZIKV Asian genotype, and (**G**) ZIKV African genotype.

Clearly SGEB provides a barrier to transmission in mosquitoes with infected salivary glands. Of all viruses tested, DENV2 American had the lowest transmission rate (11.5%), while ZIKV African had the highest (67.9%) (Table 1). As with SGIB, average viral titer (log_10_(PFU/mL)) per saliva were calculated for all seven viral genotypes, and are shown in Figure 5B. When comparing genotypes across the same virus family genotypes, titer means for DENV2, CHIKV, and ZIKV were significantly different (DENV2, *p* <0.0001; CHIKV, *p* = 0.0129; ZIKV, *p* < 0.0001). Of all viruses tested, DENV2 American had the lowest average virus titer (0.2442 ± 0.039 log_10_ (PFU/mL)), while ZIKV African had the highest titer (1.538 ± 0.1302 log_10_ (PFU/mL)) in saliva. DENV2 Cosmopolitan had the highest virus titer (0.6324 ± 0.071 log_10_ (PFU/mL)) of the three DENV2 genotypes tested, while DENV2 Asian (0.4038 ± 0.055 log_10_ (PFU/mL)) was the second lowest of all viruses tested. CHIKV ECSA-IOL had higher titers (0.922 ± 0.085 log_10_ (PFU/mL)) as compared to CHIKV Asian (0.6191 ± 0.0.079 log_10_ (PFU/mL)). CHIKV Asian had the same titer as DENV2 Cosmopolitan (*p* = 0.9013). ZIKV Asian and CHIKV ECSA-IOL both had similar titers (*p* = 0.7030) (Figure 5B). Moreover, the variance among sire means in log_10_ (PFU/mL)/saliva was not significantly different between all three DENV2 genotypes tested (American, *p* = 0.1552; Asian, *p* = 0.1945; and Cosmopolitan, *p* = 0.136) (Figure 8A–C). In contrast, the variance among sire means log_10_ (PFU/mL)/saliva for CHIKV and ZIKV genotypes were all significantly different (CHIKV ECSA-IOL, *p* = 0.0154; CHIKV Asian, *p* = 0.0242; ZIKV Asian, *p* = 0.0173; and ZIKV African, *p* = 0.0173) (Figure 8D–F).

Narrow-sense heritabilities (h^2^) for SGEB were also calculated to characterize each viral infection as SGIB. The observational components σ^2^_S_, σ^2^_d_, and σ^2^_w_ and the causal components of variance (Vp, Vsire, Vdam, Ve) for log_10_ (PFU/mL)/saliva were obtained (Table S2). In contrast to SGIB, the heritabilities for SGEB (log_10_ (PFU/mL)/saliva) were not significant for any of the three DENV2 genotypes tested (American, h^2^ = 0.319, *p* = 0.127; Asian, h^2^ = 0.160, *p* = 0.185; Cosmopolitan, h^2^ = 0.2888, *p* = 0.1103). Except for ZIKV African, the heritabilities for SGEB were likewise significant (for CHIKVECSA-IOL, h^2^ = 0.461, *p* = 0.00041; for CHIKV Asian, h^2^ = 0.437, *p* = 7.7932 × 10^−5^; for ZIKV Asian, h^2^ = 0.3286, *p* = 0.000226; and for ZIKV African, h^2^ = 0.4050, *p* = 0.0415). Clearly, genetic factors in the mosquito do influence the amount of virus in the saliva for CHIKV and ZIKV Asian (Table 3).

In examining Figure 8, note the positive trend (R = 0.86) between the variance among sire means and the h^2^_._ The average log_10_ (PFU/mL) virus in the saliva was not correlated with heritability (R = 0.552, *p* = 0.2557). Once the virus has infected the salivary gland, SGEB appears to have a major effect on the presence or absence of virus in the saliva (Table 1). SGEB has a moderate genetic basis for Zika or chikungunya infections, but not with dengue virus infections.

### 3.4. Relationship between SGIB and SGEB

We tested for a statistical correlation between log_10_ (PFU/mL)/salivary gland and log_10_ (PFU/mL)/saliva to determine whether the amount of virus in the salivary glands affected the amount of virus in the saliva. We calculated Pearson’s correlation coefficients between the log_10_ (PFU/mL)/salivary gland and the average log_10_ (PFU/mL)/saliva across all individual mosquitoes (Table 4) for all viruses tested. These correlations were only calculated among mosquitoes with virus in the salivary gland. Three of the seven genotypes have a significant correlation, but the magnitude of the correlations was small (DENV2 Asian, *p* = 0.0487; CHIKV ECSA-IOL1, *p* = 0.0026; ZIKV African, *p* < 0.0001).

An additional correlation analysis was completed between the average log_10_ (PFU/mL)/salivary gland and the average log_10_ (PFU/mL)/saliva across all sire families for a genotype. When combined across all genotypes, a correlation of R = 0.6533 (*p* < 0.0001) was obtained.

However, significant correlation was only obtained for DENV2 American (*p* = 0.0095), DENV2 Asian (*p* = 0.0124) and ZIKV African (*p* = 0.0016). This suggests that the overall correlation is an artifact of combining results from different viral genotypes. In general, the titer of virus in the salivary glands was not strongly correlated with the titer of virus in the saliva.

### 3.5. Relationship between Saliva Volume, Virus Titer, and Prevalence

To evaluate whether we were examining the genetics of SGEB underlying the amount of virus in the saliva or the SGEB genetics underlying the amount of saliva produced by mosquito, an additional 100 mosquitoes were intrathoracially injected with 500 PFU/mL for each virus, and at the time of saliva collection. All samples were assigned categories (1+, 2+, 3+, etc.) based on observed volume (Figure 2), using the stereoscope Olympus SZH10 at 2X magnification. Mosquitoes that did not visibly salivate or samples that were too difficult to measure were eliminated. Techniques have been designed to quantify saliva [47,48], but they prevent subsequent work with collected virus, and these techniques require excessive standardization to correlate the amount of label protein with the volume of saliva. We are aware this method did not (or could not) accurately determine the amount of saliva collected from individual mosquitoes, but it gave a basis for correlating the volume of saliva to the virus titer.

**Figure 9:**
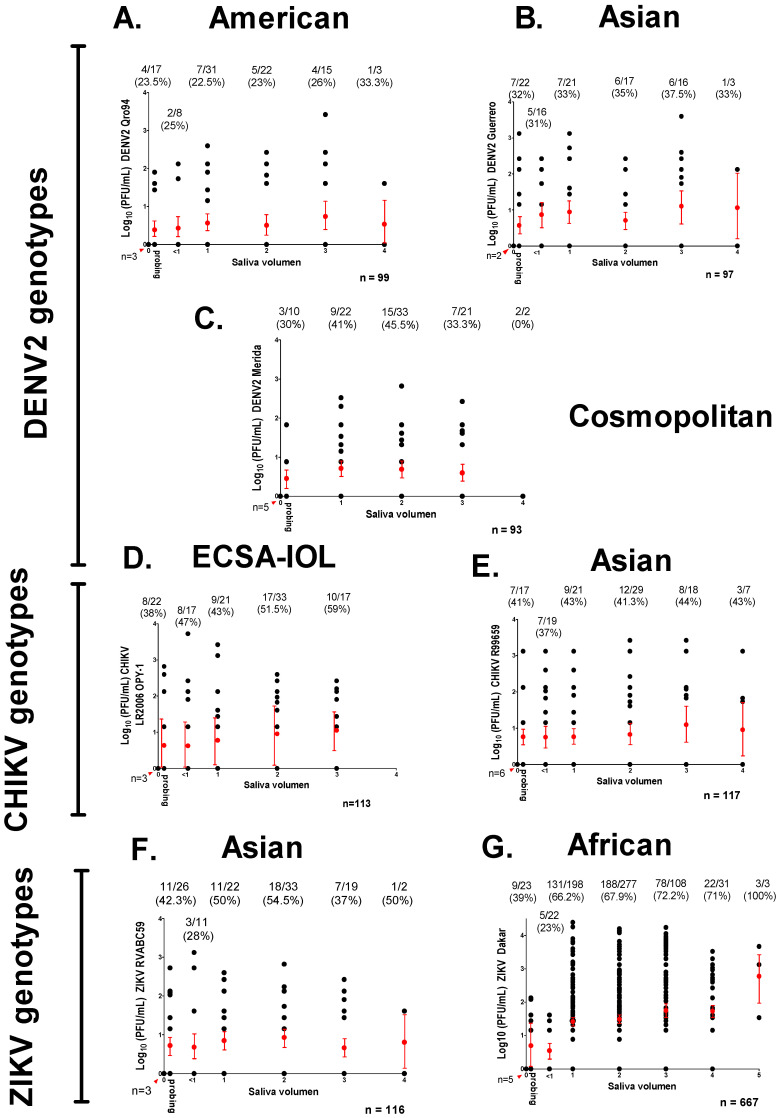
Correlation between virus titer log_10_ (PFU/mL) per saliva and volume of saliva for the seven virus strains. Each column of points arising from the X-axis represents a volume of saliva assigned. In each column, the distribution of individual saliva titers appears as black circles, also the number of positive saliva/total number of saliva per volume and prevalence is in parenthesis. Mean titer values and their 95% confidence intervals are displayed in red. Red arrow indicates non-salivated samples (*n* = 5). Total number of saliva samples tested and *p* value per virus strain are indicated. (**A**) DENV2 American genotype, (**B**) DENV2 Asian genotype, (**C**) DENV2 Cosmopolitan genotype, (**D**) CHIKV ESCA-IOL genotype, (**E**) CHIKV Asian genotype, (**F**) ZIKV Asian genotype, and (**G**) ZIKV African genotype.

If all mosquitoes produce the same concentration of virus, then we would expect the amount of virus to be proportional to the amount of saliva. The amount of live virus would also be a function of the volume of saliva released into the host, which is also a continuous variable. Alternatively, if the saliva contains clusters of viruses, then there should be no relationship between the amount of saliva and the amount of virus. It has been reported that saliva often contains clusters of viruses rather than a uniform distribution [3,22]. There is no significant difference between the amount of saliva and the virus titer (log_10_ (PFU/mL)) for all virus strains tested in this study (DENV2 genotypes: *p* = 0.8530 for American, *p* = 0.7188 for Asian, and *p* = 0.7532 for Cosmopolitan; CHIKV genotypes: *p* = 0.4236 for ECSA-IOL and *p* = 0.5910 for Asian; ZIKV genotypes: *p* = 0.7377 for Asian and *p* = 0.0963 for African). It is very clear that the amount of virus in saliva varies greatly in samples of the same volume (Figure 9). Interestingly, the prevalence of infected saliva was similar across all volumes of saliva collected (DENV2 genotypes: df = 0.1073, *p* = 0.9909 for American; df = 0.2005, *p* = 0.0.9775 for Asian; df = 0.7809, *p* = 0.8540 for Cosmopolitan; CHIKV genotypes: df = 1.923, *p* = 0.5885 for ECSA-IOL; df = 2.670, *p* = 0.6145 for Asian; ZIKV genotypes: df = 3.522, *p* = 0.3179 for Asian and df = 1.309, *p* = 0.8599 for African (chi squares values were only calculated without highest volume of saliva due to small number of samples)). This is true for all DENV, CHIKV, and ZIKV genotypes tested.

An additional correlation analysis was completed between the log_10_ (PFU/mL)/saliva and saliva volume for all viruses (Table 5). These correlations were only calculated in saliva samples positive for virus. The only significant correlation that was obtained was for ZIKV African, but the magnitude of the correlation was small (R = 0.1506, *p* = 0.0018). In general, saliva titers were not correlated with saliva volumes.

## 4. Discussion

Herein, we report the results of a half-sib quantitative genetic analysis of SGIB and SGEBs in a freshly colonized strain of *Ae. aegypti* from Mapastepec, Mexico. Half-sib analysis allows for an assessment of whether there is a genetic basis for a phenotypic trait that varies in expression in nature or in the laboratory. The critical question addressed by this study is whether there is a genetic basis for SGIB and SGEB in *Ae. aegypti*. The answer is affirmative, but with the qualification that the heritability of these traits in the mosquito is dependent upon the viral genotype, or more specifically, on the growth rate of the virus in the salivary gland. In addition, many of the significant h^2^ estimates are small, suggesting that most of the phenotypic variation is still unexplained, and that many uncontrolled factors in the environment contribute to the Vp exhibited in SGIB and SGEB. For each half-sib analysis, we assumed that the variance contributed by the virus should be minimal, because high-passaged virus in cell culture were used in this study. Usually there is abundant genetic variation present in *Ae. aegypti* field collections [25,49], and this genetic variation may affect the growth of the virus in the mosquito’s salivary gland. Examining Figure 6A,B, it is very clear that different half-sib families vary greatly in the amount of virus in salivary glands. The high h^2^ suggests that this is due to the segregation of genetic factors that affect viral reproduction among and within half-sib families. In contrast with this idea, a DENV2 genotype (Cosmopolitan) and a CHIKV genotype (Asian) (Figure 6C,E) exhibited obviously less variation in the amount of virus and higher average viral titers (Figure 5A), and the h^2^ was close to zero. Table 2 indicates significant h^2^ values for DENV2 American and Asian genotypes, for CHIKV ECSA-IOL genotype, and for ZIKV Asian genotype. Thus, it would appear that an SGIB following the introduction of virus into the hemocele has a minimal effect on salivary gland infection rates. However, SGIB did affect the quantity of virus present in the salivary gland.

A low percentage of mosquitoes injected with DENV2 genotypes remained uninfected, but all mosquitoes injected with CHIKV or ZIKV genotypes were infected (Table 1). The extrinsic incubation period varies among virus genotype and mosquito strain, and it is greatly dependent on environmental factors, such as temperature and effective dose of virus [50,51,52]. However, a minimum of one week after initial infection is required for DENV to be released and detected in saliva, indicating an absence of SGEB and SGIB [51]. There is minimal information on relative growth rates of different arboviruses following intrathoracic inoculation in mosquitoes. Since we inoculated all mosquitoes with the same number of PFU, and since we collected all mosquitoes at 7 dpi, our observations suggest, for example, that DENV2 American genotype grows more slowly than the other genotypes and that ZIKV African genotype grows faster (Figure 4). To formally test this hypothesis, we processed salivary glands and saliva from DENV2 American infected mosquitoes at a later extrinsic incubation period (14 dpi). Average virus titers in salivary glands at 14 dpi (2.6 × 10^3^ PFU/mL) were significantly higher (*p* = 0.0003) than at 7 dpi (1.7 × 10^4^ PFU/mL). However, the percentage of salivary glands infected and the percentage of saliva infected was similar, at 7 dpi and 14 dpi.

One could also vary the amount of virus injected for ZIKV and CHIKV and then assess genetic contributions throughout the course of infection. We assumed that if the variances in SGIB and SGEB are due to one or all of the random causes listed in the Introduction (such as virus genotype or limit of detection of virus by plaque assay), then Ve will be large, Va will be small, and h^2^ will approach zero. The distribution of phenotypes among the progeny of half-sib families will appear as in Figure 6C,E. Alternatively, if the amount of virus detected in salivary glands or from saliva is due, at least in part, to genetic factors, then the variation in SGIB and SGEB will have a significant h^2^. The distribution of phenotypes among the progeny of half-sib families appear as in Figure 6A,B,D,F,G. Once the virus has infected the salivary gland, SGEBs appear to have a major effect on the presence or absence of virus in the saliva (Table 1, Figure 8) and the concentration of virus in the saliva (Table 3). Saliva often contains clusters of virus rather than a uniform distribution [3,22]. Indeed, this explains the poor correlation between the amount of saliva and the amount of virus obtained for all virus strains tested in this study, as has been reported in *Ae. albopictus* infected with different CHIKV genotypes [40]. A similar argument could be made regarding the dissection of the salivary gland. It is likely that the dissected glands will be contaminated by adjacent or proximal tissues. However, if variation in the amount of virus in the dissected glands or saliva was attributable to random causes, then none of the h^2^ estimates should have been significant. However, in fact, four of the seven half-sib analyses of virus titer/salivary gland had a significant h^2^, and in three of the seven half-sib analyses of virus titer/saliva, h^2^ was significant. Thus, despite the random contribution of saliva volume and the potential presence of attached tissues during salivary gland dissections, significant heritabilities were still estimated. Further, it is unlikely that we may have underestimated h^2^ for SGEB as a result of problems associated with determining the volume of saliva produced by individual females and other uncontrolled factors. It is very clear that the amount of virus can vary greatly in similar saliva volumes (Figure 9).

The heritabilities for DENV2 SGEB were not significant, nor were the variances among sires (Figure 8, Table 3). In contrast, the heritabilities were significant, as were the variances among sires, when infected with either of the CHIKV genotypes or with ZIKV. Clearly, genetic factors in the mosquito do influence the abundance of CHIKV and ZIKV during salivation. The observed negative correlation between viral titers at the time of harvest and h^2^ (Figure 7) is consistent with a threshold model wherein once a viral population has reached a critical density, then genetic factors in the mosquito that influence viral survival and titer no longer have an effect. As the viral population approaches the threshold, the amount of variation among means of sire and h^2^ will approach zero (Figure 7). This pattern suggests that when the amount of a virus is low, genetic factors in the mosquito may influence viral titer in the salivary glands. However, once viral titers reach a high enough level in the salivary glands, the mosquito’s genetic contribution may no longer have any effect. Indeed, genetic factors associated with the innate immune response to virus infections are in play here, and the particular combination of genetic factors in a given mosquito determines how successful the virus is, so low or high titers reflect a genetic basis in the mosquito. Negatives values of Vd were obtained from DENV2 American genotype and CHIKV ECSA-IOL genotype for SGIB (Supplementary Table S1), and Vd values were negative for all six genotypes, except the DENV2 Cosmopolitan genotype, for SGEB (Supplementary Table S2). Mathematically, this occurs because the average variation virus titer (log_10_ PFU/mL) among dams within sire is less than the average variation among all sires–families. The genetic explanation for this involves either (1) dominance wherein variance among heterozygotes is not intermediate in value between parental types or (2) common environmental variance arising when progeny of a single female is all raised in the same larval container. This leads to two types of environmental variance: variance arising from a common environment (all larvae in a family raised in the same container), and variance arising from half-sibs raised under different, albeit unapparent, conditions. Because Vp = Va + Vd + Ve and h^2^ = Va/Vp, a negative dominance reduces the denominator and increases h^2^. Raising half-sib larvae in a common environment could reduce Ve and inflate the covariance among siblings, and this also could inflate h^2^. There is abundant evidence for gene by gene (G × G) interactions between arboviral genotypes and *Ae. aegypti* [53]. The genetics underlying this vector–pathogen association is still incompletely understood, but variation in vector competence appears to result, in part, from the interplay between two genomes. The findings reported here are consistent with G × G interactions. Mosquito innate immunity genes [54,55,56] impacting viral growth may only be able to act when the viral titer is low. Thus, viral genes that enable rapid growth may overcome innate immunity genes in the mosquito. In our study, variation in the mosquito arises from sampling directly from a field population, where potentially many innate immunity genes are segregating. It is clear that the different genotypes of the virus in any of the three species examined here generate a great deal of phenotypic variation in the virus propagation rates and overcome infection and transmission barriers in mosquitoes. In terms of infection rates, it is clear that SGEB provides the greatest heritable barrier to transmission in mosquitoes with infected salivary glands.

## 5. Conclusions

Herein, we report the results of a half-sib quantitative genetic analysis of SGIB and SGEBs in a freshly colonized strain of *Ae. aegypti* from Mapastepec, Mexico. The critical question addressed by this study is whether there is a genetic basis for SGIB and SGEB in *Ae. aegypti*. The answer is affirmative, but with the qualification that the heritability of these traits in the mosquito is dependent upon the viral genotype, or more specifically, on the growth rate of the virus in the salivary gland. This pattern suggests that when the amount of a virus is low, genetic factors in the mosquito may have an influence on viral titer in the salivary glands. However, once viral titers reach a high enough level in the salivary glands, the mosquito’s genetic contribution may no longer have any effect. Once the virus reaches a certain threshold, the effects of mosquito genes in determining the amount of virus declines, as does the variance in viral titers among sires. In addition, many of the significant h^2^ estimates are small, suggesting that most of the phenotypic variation is still unexplained, and that many uncontrolled factors in the environment contribute to the Vp exhibited in SGIB and SGEB.

## Figures and Tables

**Table 1:** Infection rates for salivary gland infection barrier (SGIB) and salivary gland escape barrier (SGEB) for each of the seven viral strains.

Viral Species	DENV2 Qro94	DENV2 Guerrero	DENV2 Merida	CHIKV LR 2006-OPY1	CHIKV R99659	ZIKV PRVABC-59	ZIKV Dakar 41525
**Genotype**	American	Asian	Cosmopolitan	ECSA-IOL	Asian	Asian	African
**Number of sire families**	24	21	21	22	22	21	21
**Number paired samples**	691	667	681	686	735	783	623
**Uninfected** **Glands**	38	5	4	0	0	0	0
**SGIB%**	5.50%	0.75%	0.59%	0.00%	0.00%	0.00%	0.00%
**Uninfected** **Saliva**	578	480	404	327	452	408	200
**Infected** **Saliva**	113	187	277	358	282	375	423
**SGEB%**	88.5%	72.5%	59.7%	47.7%	61.5%	52.1%	32.1%
**Transmission**	11.5%	27.5%	40.3%	52.3%	38.5%	47.9%	67.9%

**Table 2:** Quantitative genetic analysis of salivary gland infection barrier in *Aedes aegypti*. Each analysis corresponds to one of the seven virus strains tested for log_10_(PFU)/salivary gland. We used PROC MIXED in SAS to obtain restricted maximum likelihood (REML) to estimate quantitative genetic parameters. The greater the variance in sire means, the larger the heritability.

SGIB				
Virus	Strain	Genotype	h^2^	σ^2^_s_
DENV-2	Qro94	American	0.8103 ***	0.2700 **
	Guerrero C932	Asian	0.5799 **	0.1029 *
	Merida BC17	Cosmopolitan	0.0685	0.0082
CHIKV	LR2006-OPY-1	ECSA-IOL	0.5174 ***	0.0337 **
	R99659	Asian	0.0385	0.0012
ZIKV	PRVABC59	Asian	0.1526 *	0.0077
	Dakar 42525	African	0.9808 ***	0.0762 **

σ^2^s = average variance among offspring of a single sire; h^2^ = narrow-sense heritability (h^2^ = Va/Vp). * *p* = 0.03–0.05, ** *p* = 0.001, *** *p* < 0.00001.

**Table 3:** Quantitative genetic analysis of SGEB in *Aedes aegypti*. Each analysis corresponds to one of the seven virus strains tested for log_10_(PFU)/saliva. We used PROC MIXED in SAS to obtain restricted maximum likelihood (REML) to estimate quantitative genetic parameters. The greater the variance in sire means, the larger the heritability.

SGEB				
Virus	Strain	Genotype	h^2^	σ^2^_s_
DENV-2	Qro94	American	0.3186	0.0287
	Guerrero C932	Asian	0.16	0.0110
	Merida BC17	Cosmopolitan	0.2888	0.0258
CHIKV	LR2006-OPY-1	ECSA-IOL	0.4611 ***	0.0914 *
	R99659	Asian	0.4372 ***	0.1025 *
ZIKV	PRVABC59	Asian	0.3286 ***	0.0839 *
	Dakar 42525	African	0.4050	0.1569

σ^2^_s_ = average variance among offspring of a single sire; h^2^ = narrow-sense heritability (h^2^ = Va/Vp). * *p* = 0.01, *** *p* < 0.0001.

**Table 4:** Correlation analysis between the log10 (PFU/mL)/salivary gland and the log_10_ (PFU/mL)/saliva across all individual mosquitoes for each of the seven genotypes. Mosquitoes without an infected salivary gland were not included. *p* values that are bolded are significant at a threshold of *p* < 0.05.

Virus Strain	Genotype	Corr. Coeff. (R)	Prob. (*p*)
DENV2 Qro94	American	−0.0503	0.6003
DENV2 GuerreroC932	Asian	0.1444	**0.0487**
DENV2 Merida BC17	Cosmopolitan	0.0255	0.6726
CHIKV R99659	Asian	0.0649	0.2774
CHIKV LR2006OPY-1	ECSA-IOL	0.1591	**0.0026**
ZIKV PRVABC59	Asian	−0.0453	0.2118
ZIKV Dakar	African	0.1947	**<0.0001**

**Table 5:** Correlation analysis between the log_10_ (PFU/mL)/saliva and saliva volume for each virus. *p* values that are bolded are significant at a threshold of *p* < 0.05.

Virus Strain	Genotype	Corr. Coeff. (R)	Prob. (*p*)
DENV2 Qro94	American	0.1258	0.5001
DENV2 GuerreroC932	Asian	0.1528	0.3596
DENV2 Merida BC17	Cosmopolitan	0.1570	0.3466
CHIKV R99659	Asian	0.1856	0.1876
CHIKV LR2006OPY-1	ECSA-IOL	0.0924	0.4981
ZIKV PRVABC59	Asian	−0.0384	0.7728
ZIKV Dakar	African	0.1506	**0.0018**

## Data Availability

All data produced from this study are included in this published paper.

## Supplementary Materials

The following are available online at https://www.mdpi.com/2075-4450/12/1/73/s1, Figure S1: The SAS code for performing REML analyses, Table S1: Quantitative genetic analysis of SGIB in *Aedes aegypti*, Table S2. Quantitative genetic analysis of SGEB in *Aedes aegypti*.
