# Supplementary material for: The Genetic Basis for Salivary Gland Barriers to Arboviral Transmission
Source: Insects. 2021 Jan 15;12(1):73. doi: 10.3390/insects12010073 (PMC7830681; doi:10.3390/insects12010073)
Supplement: Supplementary file 1 [file insects-12-00073-s001.zip › supplementary-xml/Figure S1.pdf]

```
DATA ZIKV;
INPUT progeny sire dam orig tvalue trait$;
CARDS;
        1         41          4     660000       5.820          SG
        1         41          4       19.8       1.318         Sal
        2         41          4     264000       5.422          SG
        2         41          4          0       0.000         Sal
;
        */ Salivary glands pfu;
        PROC MIXED data = ZIKVcovtest asycov;
        class sire dam;
        where TRAIT = "SG";
        model tvalue = /solution;
        random Sire Dam(Sire);
        run;
        PROC MIXED data = ZIKVcovtest asycov;
        class sire dam;
        where TRAIT = "SG";
        model tvalue = /solution;
        random dam(sire);
        run;
        PROC MIXED data = ZIKVcovtest asycov;
        class sire dam;
        where TRAIT = "SG";
        model tvalue = /solution;
        random sire;
        run;
        */ Saliva pfu;
        PROC MIXED data = ZIKVcovtest asycov;
        class sire dam;
        where TRAIT = "Sal";
        model tvalue = /solution;
        random Sire Dam(Sire);
        run;
        PROC MIXED data = ZIKVcovtest asycov;
        class sire dam;
        where TRAIT = "Sal";
        model tvalue = /solution;
        random Dam(Sire);
        run;
        PROC MIXED data = ZIKVcovtest asycov;
        class sire dam;
        where TRAIT = "Sal";
        model tvalue = /solution;
        random Sire;
        run;
```

Figure S1. The SAS code for performing REML analyses.
