# Supplementary material for: The Genetic Basis for Salivary Gland Barriers to Arboviral Transmission
Source: Insects. 2021 Jan 15;12(1):73. doi: 10.3390/insects12010073 (PMC7830681; doi:10.3390/insects12010073)
Supplement: Supplementary file 1 [file insects-12-00073-s001.zip › supplementary-xml/Supplementary Tables.pdf]

Table S1. Quantitative genetic analysis of SGIB in *Aedes aegypti.*

	DENV2Qro94 American				DENV2 Guerrero C932 Asian				DENV2 Merida BC17 Cosmopolitan			
	Estimate	Error	*z*-value	Prob>*z*	Estimate	Error	*z*-value	Prob>*z*	Estimate	Error	*z*-value	Prob>*z*
σ^2_s	0.2700	0.0975	2.7700	0.0028	0.1029	0.0567	1.8100	0.0348	0.0082	0.0179	0.4600	0.3238
σ^2_d	0.0479	0.0300	1.5900	0.0555	0.12	0.0382	3.1400	<0.0001	0.0833	0.0264	3.1600	0.0008
σ^2_w	1.0149	0.0546	18.6000	<0.0001	0.4869	0.0762	17.6300	<0.0001	0.3875	0.0219	17.7100	<0.0001
σ^2_p	1.3328				0.7098				0.4789			
Va	1.0800				0.4116				0.0328			
Vd	−0.2221				0.0171				0.0751			
Ve	0.4749				0.2811				0.3711			
Vp	1.3328				0.7098				0.7489			
h^2	**0.8103**				**0.5799**				**0.0685**			
prob $h^2 = 0$	5.34E−07				0.0072				0.3274			
	CHIKV LR2006 OPY-1 ECSA-IOL				CHIKV R99659 Asian							
	Estimate	Error	*z*-value	Prob>*z*	Estimate	Error	*z*-value	Prob>*z*				
σ^2_s	0.0337	0.0131	2.5800	0.005	0.0012	0.0102	0.1200	0.4537				
σ^2_d	0.0017	0.0049	0.3400	0.3562	0.0072	0.0128	0.5600	0.2882				
σ^2_w	0.2248	0.0127	17.7500	<0.0001	0.1155	0.0204	5.6700	<0.0001				
σ^2_p	0.2601				0.1238							
Va	0.1346				0.0048							
Vd	−0.0320				0.006							
Ve	0.1575				0.1131							
Vp	0.2601				0.1238							
h^2	**0.5174**				**0.0385**							
prob $h^2=0$	6.20E−06				0.5000							
	ZIKV PRVABC59 Asian				ZIKV Dakar African							
	Estimate	Error	*z*-value	Prob>*z*	Estimate	Error	*z*-value	Prob>*z*				
σ^2_s	0.0077	0.00558	1.3300	0.0918	0.0762	0.343	2.2200	0.0133				
σ^2_d	0.0219	0.0072	3.05	0.0012	0.0466	0.0159	3.05	0.0011				
σ^2_w	0.1719	0.009	19.11	<0.0001	0.1859	0.011	16.84	<0.0001				
σ^2_p	0.2015				0.3106							
Va	0.0307				0.0305							
Vd	0.0142				−0.0276							
Ve	0.1565				0.0336							
Vp	0.2015				0.3106							
h^2	**0.1526**				**0.9808**							
prob $h^2 = 0$	4.16E−02				0.0009							

Table S2. Quantitative genetic analysis of SGEB in *Aedes aegypti.*

	DENV2Qro94 American				DENV2 Guerrero C932 Asian				DENV2 Merida BC17 Cosmopolitan			
	Estimate	Error	*z*-value	Prob>*z*	Estimate	Error	*z*-value	Prob>*z*	Estimate	Error	*z*-value	Prob>*z*
σ^2_s	0.0287	0.0283	1.0100	0.1552	0.011	0.0127	0.8600	0.1945	0.0258	0.0235	1.1000	0.136
σ^2_d	0.0015	0.0353	0.0400	0.4829	0.0000	0.0000	0.0000	0.0000	0.0319	0.0263	1.2100	0.1126
σ^2_w	0.3300	0.0511	6.4600	<0.0001	0.2631	0.0283	9.2900	<0.0001	0.2996	0.0289	10.4700	<0.0001
σ^2_p	0.3602				0.2741				0.3573			
Va	0.1148				0.0438				0.1032			
Vd	–0.0272				–0.011				0.0061			
Ve	0.2726				0.2412				0.2480			
Vp	0.3602				0.2741				0.3573			
h^2	**0.3186**				**0.1600**				**0.2888**			
prob h^2 = 0	0.1271				0.1855				0.1103			
	CHIKV LR2006 OPY-1 ECSA-IOL				CHIKV R99659 Asian							
	Estimate	Error	*z*-value	Prob>*z*	Estimate	Error	*z*-value	Prob>*z*				
σ^2_s	0.0914	0.0423	2.1600	0.0154	0.1025	0.052	1.9700	0.0242				
σ^2_d	0.0649	0.0256	2.5300	0.0057	0.1024	0.0381	2.6900	0.0036				
σ^2_w	0.6363	0.0347	18.3400	<0.0001	0.7328	0.0414	17.7200	<0.0001				
σ^2_p	0.7926				0.9377							
Va	0.3655				0.41							
Vd	–0.0265				–0.0001							
Ve	0.4536				0.5278							
Vp	0.7926				0.9377							
h^2	**0.4611**				**0.4372**							
prob h^2 = 0	0.0004				0.0001							
	ZIKV PRVABC59 Asian				ZIKV Dakar African							
	Estimate	Error	*z*-value	Prob>*z*	Estimate	Error	*z*-value	Prob>*z*				
σ^2_s	0.0839	0.0397	2.1100	0.1730	0.1569	0.1118	1.4000	0.0804				
σ^2_d	0.0710	0.0292	2.3800	0.0086	0.3233	0.1017	3.1800	0.0007				
σ^2_w	0.8664	0.0457	18.96	<0.0001	1.0675	0.0634	16.8400	<0.0001				
σ^2_p	1.0213				1.5476							
Va	0.3356				0.6272							
Vd	–0.0129				–0.1665							
Ve	0.6986				0.7539							
Vp	1.0213				1.5476							
h^2	**0.3286**				**0.4050**							
prob h^2 = 0	0.0002				0.0830							
